# Ibrutinib in Elderly Patients with Chronic Lymphocytic Leukemia: Adverse Event Incidence, Management, and Outcomes in a Canadian Real-World Setting

**DOI:** 10.3390/curroncol32060296

**Published:** 2025-05-23

**Authors:** Ibraheem Othman, Seyedeh Zahra (Mona) Moossavi, Samaneh Bayati, Yi Sin Chang, Shubrandu Sanjoy, Karolina Grzyb, Eric Sy, Kayla Cropper, Sandy Kassir, Waleed Sabry

**Affiliations:** 1 Division of Hematology, Department of Medical Oncology and Hematology, Allan Blair Cancer Centre, Regina, SK S4T 7T1, Canada; 2 College of Medicine, University of Saskatchewan, Saskatoon, SK S7N 5E5, Canada; mona.moossavi@usask.ca (S.Z.M.); samaneh.bayati@usask.ca (S.B.); yisin.chang@usask.ca (Y.S.C.); eric.sy@saskhealthauthority.ca (E.S.); kayla.cropper@usask.ca (K.C.); waleed.sabry@saskcancer.ca (W.S.); 3 Epidemiology and Performance Measurement, Saskatchewan Cancer Agency, Saskatoon, SK S7N 4H4, Canada; shubrandu.sanjoy@saskcancer.ca; 4 Research Department, Saskatchewan Health Authority, Regina, SK S7K 0M7, Canada; karolina.grzyb@health.gov.sk.ca (K.G.); sandy.kassir@saskhealthauthority.ca (S.K.); 5 Department of Critical Care, Saskatchewan Health Authority, Regina, SK S4P 0W5, Canada; 6 Division of Hematology, Department of Medical Oncology and Hematology, Saskatoon Cancer Centre, Saskatoon, SK S7N 4H4, Canada

**Keywords:** chronic lymphocytic leukemia, ibrutinib, adverse events, treatment discontinuation

## Abstract

Background: Long-term clinical trials and real-world data have established a comprehensive risk–benefit profile for ibrutinib, informing adverse event (AE) management strategies to optimize safety and efficacy. Methods: We retrospectively assessed the incidence of AEs of special interest and management strategies in all patients treated with ibrutinib for chronic lymphocytic leukemia (CLL) in Saskatchewan, Canada, since 2014. Results: Among 187 patients (median age 75.7 years, 63% male), the median time from ibrutinib treatment initiation to data cutoff was 3.1 years. Approximately two-thirds of patients received ibrutinib for relapsed CLL (33.7% second-line and 32.6% third-line and beyond), with 33.7% receiving it first-line. All patients initiated ibrutinib as monotherapy at 420 mg. AEs of interest were observed in 81.3% of patients, with 42.8% experiencing ≥2 AEs. No grade 5 AEs were reported. Among the 284 first-onset AEs observed in 152 patients, 90.8% were successfully managed, allowing treatment continuation. The median time to successful management ranged from 27.0 days (range: 12.5–73.0) for infections to 84.0 days (range: 55.0–141.0) for hypertension. Both AE and discontinuation rates were comparable or favourable to previous reports. Conclusion: This real-world analysis suggests that ibrutinib may be safely used in the majority of CLL patients encountered in routine practice.

## 1. Introduction

Chronic lymphocytic leukemia (CLL) is one of the most common types of adult leukemia in Canada, with over 2200 new cases diagnosed each year [1,2]. The first-in-class Bruton tyrosine kinase inhibitor (BTKi) ibrutinib was authorized by Health Canada in 2014 due to its transformative efficacy in treating adult patients with relapsed/refractory CLL [3]. At that time, it represented the first once-daily, orally administered treatment for the disease. Subsequently, in 2016, ibrutinib was approved for use in patients with previously untreated CLL. Since then, it has received approval for use across all lines of CLL therapy and in various combinations [4]. A large retrospective analysis conducted in British Columbia, involving medical records of over 1700 patients diagnosed with CLL between 1984 and 2016, demonstrated improved overall survival (OS) over time following the introduction of ibrutinib [5]. Two-year OS rates were 95.7%, 88.0%, and 84.9% for patients receiving ibrutinib as first-line, second-line, and third-line or later therapy, respectively [6]. In addition, a separate Canadian study confirmed the effectiveness of ibrutinib in an ethnically diverse community hospital population [7].

Long-term clinical trials [8,9,10,11,12] and real-world data [13,14,15,16] have helped to develop a comprehensive risk–benefit profile, informing strategies for managing adverse events (AEs) and optimizing both patient safety and treatment outcomes. However, reports of serious AEs [16,17,18,19,20] and recent head-to-head trials [21,22] comparing next-generation BTKis with ibrutinib have raised concerns about its toxicity and ongoing use in clinical practice.

Despite ibrutinib being available for over a decade, real-world data on its use in routine Canadian practice, particularly regarding the occurrence and management of treatment-related toxicities, still need to be explored. To address this gap, we conducted a comprehensive assessment of AEs of special interest associated with ibrutinib treatment in patients diagnosed with CLL in Saskatchewan. Our focus was on management strategies and subsequent outcomes, specifically the resolution of AEs and patients’ ability to continue ibrutinib therapy. This evaluation aimed to provide deeper insights into ibrutinib’s safety profile and the effectiveness of various management approaches used in our clinical practice, where most patients tend to be older and/or have comorbidities, in contrast to those typically enrolled in controlled clinical trials.

Further characterization of ibrutinib-related AEs and their management in such populations is particularly important, especially following the recent Health Canada approval for its use in combination with venetoclax. This combination offers a novel, fixed-duration, all-oral treatment approach, which may provide both convenience for CLL patients and cost savings to the Canadian healthcare system. By analyzing the frequency of AEs of special interest and their management, we aim to enhance our understanding of how ibrutinib is utilized in clinical settings and identify best practices for managing potential AEs that may arise during treatment.

## 2. Methods

### 2.1. Data Collection

This retrospective cohort study involved a review of medical charts of adult patients treated with ibrutinib for CLL in Saskatchewan at the Allan Blair Cancer Centre or the Saskatoon Cancer Centre between 2014 and 2023. The inclusion criteria included the following:

- Adult patients (≥18 years old) with confirmed CLL according to the International Workshop on CLL (iwCLL; 2018) criteria;
- Documented use of ibrutinib as frontline or relapsed/refractory treatment, either as monotherapy or in combination therapy;
- Known ibrutinib start date;
- ≥12 months of follow-up from initiation of treatment with ibrutinib.

Given that cytogenetic and mutational testing were not routinely performed at the time of study initiation, these data were not collected.

AEs were defined as new events occurring during ibrutinib treatment, and were identified through a review of available hospital and emergency department records.

We reported the incidence of AEs of interest, including anemia, arthralgia, atrial fibrillation (AF), diarrhea, hypertension, infection, major bleeding, neutropenia, rash, and thrombocytopenia, that occurred while patients were actively receiving ibrutinib. Infections were documented by treating clinicians based on clinical indicators such as fever, changes in leukocyte counts, and organ-specific symptoms; however, information on infection type or pathogen was not collected. Hematological AEs were defined and graded based on the criteria established by the iwCLL, while non-hematological AEs were defined and graded according to the National Cancer Institute’s Common Terminology Criteria for Adverse Events (CTCAE, version 5.0). Grading was determined either by direct provider entry or retrospectively assigned by the principal investigator based on available chart documentation.

### 2.2. Outcomes of Interest

The following outcomes were collected: the time to onset of AEs of interest and the time required for successful management of these AEs; the frequency of ibrutinib dose modifications (interruptions and reductions) associated with each AE; the use of additional therapies to address AEs; the rate of treatment discontinuation due to AEs; and the proportion of successfully managed AEs during the time on ibrutinib.

Dose interruptions were categorized as follows: a dose hold for ≤1 month, a dose hold for 2 months, and a dose hold for 3 to 12 months. Dose reductions were defined as a reduction from 420 mg to 280 mg (from 3 to 2 tablets), from 420 mg to 140 mg (from 3 to 1 tablet), from 280 mg to 140 mg (from 2 to 1 tablet), and to doses less than 140 mg. Dose adjustments were made at the discretion of the treating physician, and generally followed the recommendations outlined in the product monograph.

Additional therapies to manage AEs were classified as follows: symptomatic (e.g., medications to treat the event), supportive (e.g., medications and/or IV fluids, blood products), and vigorous supportive (e.g., requiring surgery or intubation). Management strategies were tailored to AE severity, with more severe cases requiring intensive approaches that often combined symptomatic and supportive measures.

The assessment of the management response to an AE was classified as either success or failure. Success was defined as resolution or control of the AE, indicated by a reduction in AE recurrence, a decrease in AE severity, or the prevention of AE worsening, allowing for continued treatment with ibrutinib. Failure was defined as treatment discontinuation or death related to the AE. The evaluation of AE responses was conducted by the Principal Investigator.

### 2.3. Statistical Analysis

All statistical analyses were performed using Stata 15.1/MP (StataCorp, College Station, TX, USA) and the latest version of RStudio and NVivo software (QSR International Pty Ltd., Melbourne, VIC, Australia, 2020). Qualitative variables were reported as counts and percentages, while quantitative variables were expressed as the mean ± standard deviation or median (interquartile range [IQR]).

## 3. Results

A total of 187 medical charts for patients treated with ibrutinib for CLL between 2014 and 2023 met the inclusion criteria. The median time from treatment initiation to data cutoff for the entire cohort was 3.1 years. Baseline demographics, the line of treatment in which ibrutinib was administered, and coexisting conditions at the initiation of ibrutinib are summarized in Table 1.

Overall, the median [IQR] age was 75.7 [66.7–82.4] years, with 63.1% (n = 118) male. Most patients received ibrutinib for the treatment of relapsed CLL (33.7% for the second line and 32.6% for the third line and beyond), with 33.7% receiving it as a frontline treatment. The most common coexisting conditions at treatment initiation were diabetes (n = 29 [15.5%]), renal disease (n = 19 [10.2%]), and chronic obstructive pulmonary disease (n = 16 [8.6%]). Approximately a quarter of patients (n = 46 [24.6%]) presented with baseline cardiovascular (CV) comorbidities or risk factors. All patients began ibrutinib as monotherapy at a dose of 420 mg daily.

### 3.1. AEs of Interest

**Figure 1:**
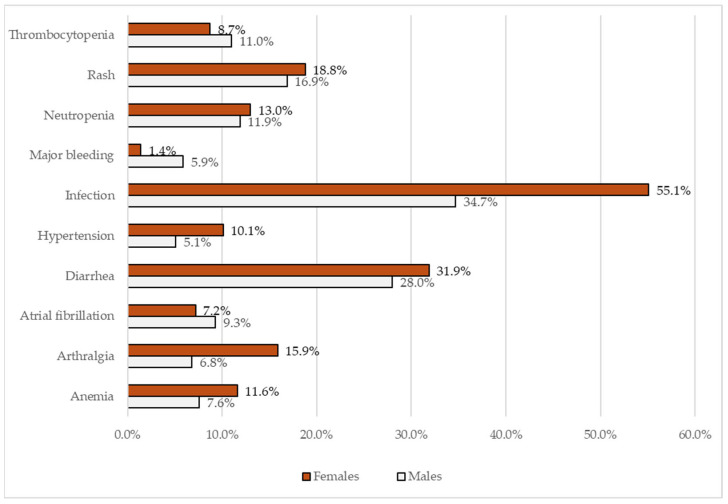
Rates of ibrutinib-related adverse events (AEs) by gender.

At the time of database lock, the median (IQR) duration of ibrutinib treatment was 2.9 (0.8–5.6) years. AEs of interest were observed in 152 patients (81.3%), with 80 patients (42.8%) experiencing ≥2 AEs. Of the 376 AE cases recorded during the study period, 284 (75.5%) were first-onset AEs, predominantly grade 1 or 2 (72.2%), with no grade 5 AEs, as shown in Table 2. There were no reported cases of ventricular arrhythmia or hepatitis reactivation. The most common AEs observed in ≥10% of patients were infections (42.8%), diarrhea (29.4%), rash (17.6%), neutropenia (12.8%), arthralgia (10.2%), and thrombocytopenia (10.2%). COVID-19 infections occurred in 16 patients (8.6%). Any-grade AF and hypertension, cardiac AEs of special interest, were recorded in 8.6% and 7.0% of patients, respectively, and were predominantly grade 1/2. Infections, arthralgia, and hypertension occurred more frequently in female patients, whereas major bleeding was observed more often in males (Figure 1).

**Figure 2:**
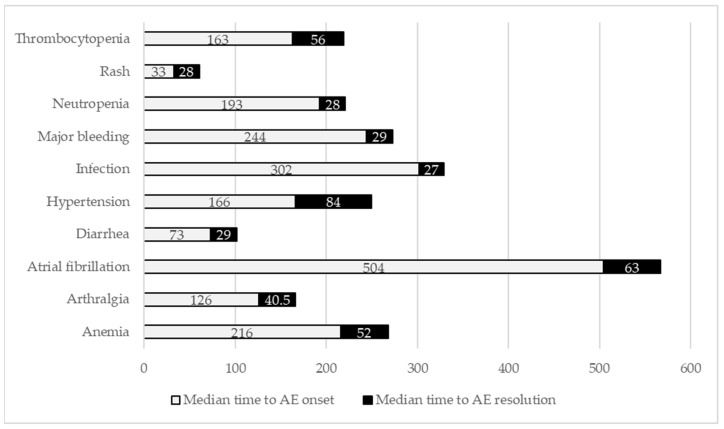
Median time to adverse event (AE) onset and resolution (days).

The median time to AE onset ranged from 33 days for rash to 504 days for AF (Figure 2). The median time to successful management varied, ranging from 27.0 days (range: 12.5–73.0) for infections to 84.0 days (range: 55.0–141) for hypertension. Except for infections (20.3% of patients experiencing a second infection and 5.3% experiencing a third), AE recurrence rates were low and occurred in less than 10% of patients (Table 2).

### 3.2. AE Management Strategies

AEs led to ibrutinib hold, dose reduction, and discontinuation in 56 (29.9%), 22 (11.8%), and 31 (16.6%) patients, respectively. Among the 63 patients who received ibrutinib as a first-line therapy, 4 (6.3%) required dose reduction due to AEs, compared with 18 (14.5%) of the 124 patients treated in the second-line setting. In general, management strategies were adapted to the severity of the AE, with more severe AEs necessitating more intensive interventions that often involved a combination of symptomatic and supportive measures.

**Figure 3:**
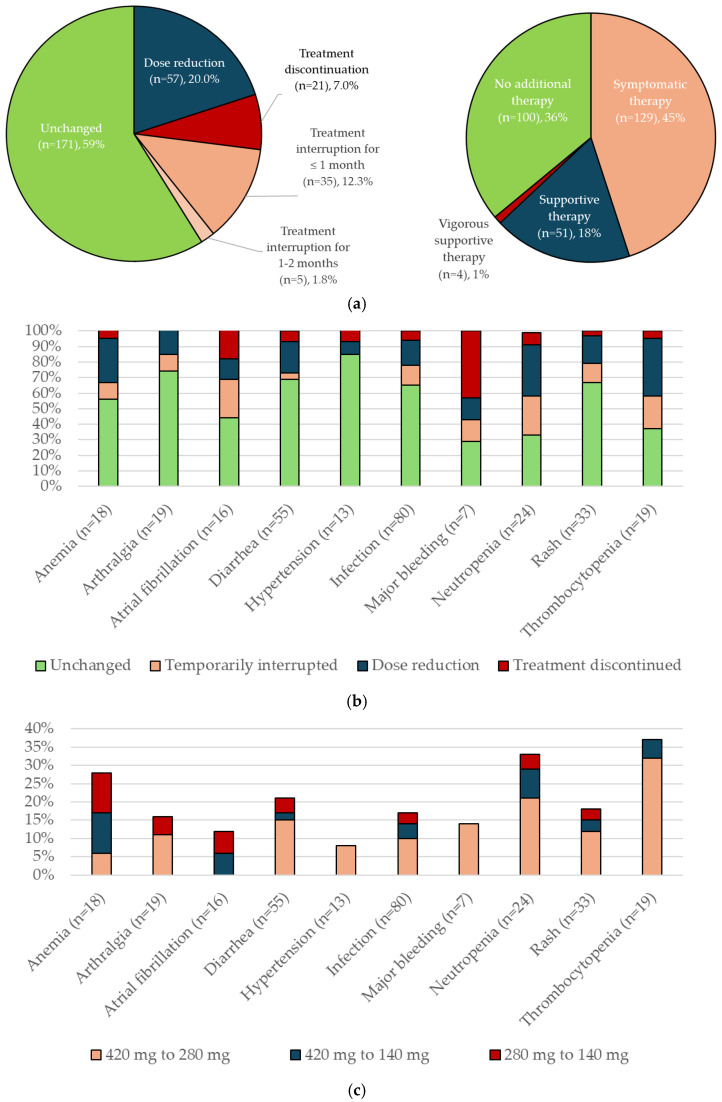

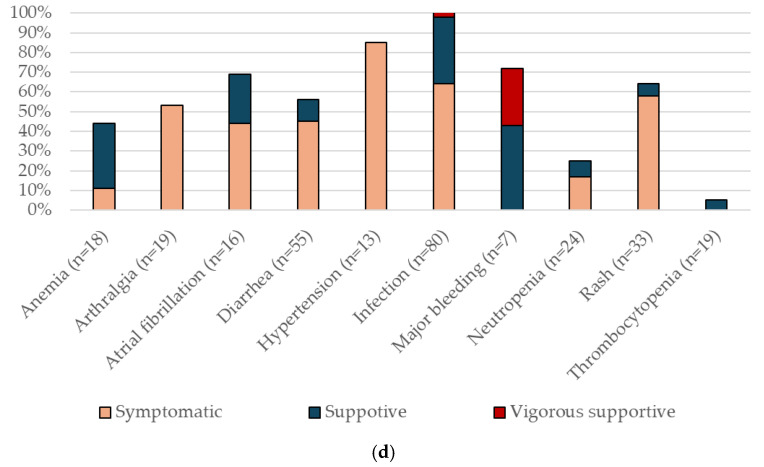
Adverse event (AE) management strategies: (**a**) first-onset AE management strategies; (**b**) dosing of ibrutinib by AE; (**c**) ibrutinib dose modifications by AE; (**d**) additional therapy to treat AEs.

Of the 284 first-onset AE cases observed in 152 patients, 90.8% were successfully managed, allowing for continued active treatment with ibrutinib. Management strategies included a dose hold of ≤1 month (12.3% of cases) or 1–2 months (1.8% of cases), followed by reinitiation of ibrutinib at the same dose (79.9%) or a lower dose (12.3% of patients were reinitiated at 280 mg and 7.7% at 140 mg); see Figure 3a. No patient received a dose lower than 140 mg. Hematologic toxicities were most likely to necessitate dose reductions (5/18 [28%] for anemia, 8/24 [33%] for neutropenia, and 7/19 [37%] for thrombocytopenia), while major bleeding was most likely to lead to treatment discontinuation (3/7 [43%] cases); see Figure 3b,c. Symptomatic therapy was required for 129/284 (45%) first-onset AEs, supportive interventions for 51/284 (18%), and vigorous supportive measures for 4/284 (1.4%); see Figure 3d. While 11/13 (85%) hypertension cases and 10/19 (53%) arthralgia cases were managed with symptomatic therapy alone, AF and infections required both symptomatic and supportive approaches (7/16 (44%) symptomatic and 4/16 (25%) supportive for AF, and 51/80 (64%) symptomatic and 27/8 (34%) supportive for infections). Major bleeding required supportive measures in 3/7 (43%) cases and vigorous supportive measures in 2/7 (29%) cases. Vigorous supportive measures were also used in 2/80 (3%) infections. For hematological toxicities, 2/18 (11%) and 4/24 (17%) patients with anemia and neutropenia, respectively, were treated with symptomatic therapy. Supportive therapy was used in 6/18 (33%) patients with anemia, 2/24 (8%) with neutropenia, and 1/19 (5%) with thrombocytopenia.

A total of 31 out of 187 patients discontinued ibrutinib due to an AE (21 after the first onset), including 3 patients (2 after the first onset) who chose to discontinue treatment. Discontinuation was most frequently necessitated for major bleeding (3/7 [43% of cases]), dose hold due to AF (4/16 [25%]) and neutropenia (6/24 [25%]), and dose reductions for thrombocytopenia (7/17 [37% of cases]).

## 4. Discussion

The first-generation BTK inhibitor ibrutinib has been characterized as having higher rates of class-related toxicities when compared to the second-generation BTK inhibitors acalabrutinib and zanubrutinib [21,22]. Although severe CV events are not unique to ibrutinib, earlier clinical trials and real-world studies reported these events more frequently with ibrutinib than with zanubrutinib and acalabrutinib [23,24,25,26,27]. However, a recent meta-analysis focusing on studies comparing ibrutinib to second-generation BTK inhibitors revealed similar risks of coronary artery disease (CAD), ventricular tachycardia, sudden cardiac death, hypertension, and treatment discontinuation due to cardiac events between ibrutinib and second generation BTKi [28]. An increase in the rates of CV toxicities with longer follow-ups has also been documented, which has been reflected in the subsequent acalabrutinib Food and Drug Administration (FDA) label and the US package insert update [29]. It is also revealed that the timing of AE onsets varies among BTKis [30], indicating that all patients treated with these therapies require CV surveillance according to their pre-treatment CV risk factors, as well as the specific BTKi prescribed.

Given ibrutinib’s continued role in CLL treatment, with nearly 200 active trials investigating its use in CLL [31] and its incorporation into various combination regimens, including the first all-oral, fixed-duration regimen with venetoclax [32,33], the successful management of ibrutinib-related AEs in our cohort warrants closer examination, as it offers valuable insights into real-world clinical practice. While it is anticipated that the AE profile of fixed-duration regimens will differ from that of continuous therapy, effective management of these AEs remains crucial for optimizing patient outcomes.

Our study is distinct in that it differs from both randomized clinical trials and most real-world analyses. While real-world studies often focus on patients treated at academic centers, many of whom have high-risk disease or comorbidities, leading to potential selection bias, our analysis includes all patients treated with ibrutinib across an entire province. This population-based approach minimizes selection bias and offers a more comprehensive perspective on ibrutinib-related AEs in an unselected CLL population. In contrast, controlled clinical trials typically enroll younger and healthier patients, with a median age of 67 years, even though the median age at CLL diagnosis in the general population is 70 years, and more than one-third of patients are over 75 years of age. These trials also frequently exclude patients with significant CV risk factors or an Eastern Cooperative Oncology Group Performance Status (ECOG PS) > 2, thereby potentially excluding patients with comorbidities. In our study, 26.2% of patients were over the age of 75, with a median age of 75.6 years, which more closely reflects the general CLL population. Additionally, many patients had baseline CV risk factors, including prior myocardial infarction (4%), congestive heart failure (2%), cerebrovascular disease (2%), and diabetes (16%). Renal disease was also present in 10% of patients.

Over a 10-year follow-up period, no patients died due to ibrutinib-related AEs. Furthermore, most AEs were manageable, resulting in significantly lower treatment discontinuation rates than those typically reported in other real-world studies. These lower discontinuation rates may reflect the proactive pre-treatment evaluation and CV risk screening implemented in our clinical practice. Each patient treated with BTK inhibitors, including ibrutinib, in Saskatchewan undergoes a detailed medical history review and a focused CV risk examination, including an electrocardiogram (ECG) and blood pressure measurement, to identify common risk factors such as AF and hypertension.

After the median follow-up of 3.1 years and the median duration of ibrutinib treatment of approximately 35 months, only 16.6% of patients treated with ibrutinib in Saskatchewan discontinued treatment due to AEs, a rate lower than that reported in other real-world studies [16,34,35] and clinical trials. For instance, in both the ELEVATE-RR and ALPINE trials, which compared ibrutinib to other Bruton’s tyrosine kinase inhibitors (acalabrutinib in ELEVATE-RR and zanubrutinib in ALPINE) in previously treated CLL, the AE-related discontinuation rates for ibrutinib were 21.3% after 35 months in ELEVATE-RR and 23% after 30 months in ALPINE [21,22]. These findings are consistent with a real-world analysis of nearly 12,000 Medicare beneficiaries, in which nearly two-thirds of patients who initiated ibrutinib discontinued treatment after a median follow-up of two years, with toxicities as a primary driver [16].

The incidence of hypertension, atrial fibrillation, major bleeding, and grade ≥ 3 neutropenia and anemia in our study aligns with an Italian multicenter retrospective study of CLL patients over 80 years of age treated with first-line ibrutinib [36]. These parallel findings may reflect similar patient selection practices and proactive risk mitigation strategies in elderly populations. This may be particularly relevant for managing hypertension, a known contributor to major adverse cardiovascular events (MACEs) associated with BTK inhibitors [37].

In a large retrospective analysis of patients with lymphoid malignancies treated with ibrutinib, nearly 20% of those who developed new or worsening hypertension experienced MACEs, compared to less than 10% among those with stable or no hypertension [34]. Although the median time to hypertension onset in our cohort was similar (5.5 months), the overall incidence of new all-grade hypertension was significantly lower (7% vs. 78%). This reduced rate may help to explain the low incidence of AF observed in our study (8.6%) and, notably, the low rate of AF-related treatment discontinuation (1%).

It is also important to note the extended time between ibrutinib initiation and the onset of some AEs in our cohort. The median time to AF onset exceeded 16 months, while infections and major bleeding occurred at median times of 10 and 8 months, respectively. The reasons for these delays are not fully understood, and warrant further investigation to determine whether they are primarily the result of outliers in a small sample size (only 16 patients developed AF, and 7 developed major bleeding), or whether underlying factors, patient selection, or pre-emptive measures may play a role. Regardless of the cause, our data indicate that ibrutinib-related AEs can develop at any time, highlighting the need for continuous vigilance and routine patient monitoring for signs of AEs.

When interpreting the results of our study, one should keep in mind the inherent data collection limitations associated with retrospective, real-world data collection. Accordingly, several limitations must be considered, including the study’s descriptive nature and the absence of an in-depth analyses to assess causal relationships between ibrutinib discontinuation (timing/status) and efficacy outcomes. Additionally, we did not identify specific coexisting factors that may have predisposed patients to AEs. For example, it was not possible to determine whether baseline comorbidities contributed to AE risk, whether bleeding events occurred in patients on anticoagulants, or whether infections were linked to neutropenia. Similarly, infection sites and causes of death unrelated to ibrutinib were not captured.

Additionally, we acknowledge that the line of therapy may influence patient-reported tolerability. In our study, two-thirds of patients had relapsed disease, and thus, the perceptions and thresholds for AE reporting may differ from those in treatment-naïve patients. This may have contributed to lower rates of reported AEs, particularly in the context of less rigorous data collection compared to clinical trials. Despite these limitations, our findings support the favorable safety profile of single-agent ibrutinib for the treatment of CLL.

Future analyses will examine and compare time to next treatment (TTNT) among patients who discontinue ibrutinib due to treatment-related AEs, those who discontinue for other reasons, and those who remain on treatment or discontinue treatment due to progression. These insights will further inform clinical decision-making and help to assess the real-world effectiveness of continuous ibrutinib therapy.

## 5. Conclusions

This real-world analysis supports the fact that ibrutinib can be safely used and effectively managed in the majority of CLL patients encountered in routine practice, including elderly patients. AE and related discontinuation rates were comparable to or more favourable than those observed in previous reports, with no AE-related deaths observed. AE management strategies that enabled continued active treatment were effective for most, but not all, AEs and patients. This highlights the need for patient-tailored approaches based on individual risk profiles and the long-term potential for additional complications. Further research is needed to better understand the population characteristics and treatment responses that can guide clinicians in the optimal management of patients with multiple comorbidities and concurrent medications who are receiving ibrutinib for CLL.

## Figures and Tables

**Table 1:** Characteristics of the study population.

Characteristics	N = 187
Age at ibrutinib initiation (median [IQR]), years	75.7 [66.7, 82.4]
≥75 years, n (%)	49 (26.2%)
Gender, n (%)	
Female	69 (36.9)
Male	118 (63.1)
Area of residence, n (%)	
Urban	147 (78.6)
Rural	29 (15.5)
Not available	11 (5.9)
Line of therapy in which ibrutinib was initiated, n (%)	
First line	63 (33.7)
Second line	63 (33.7)
Third line	40 (21.4)
Fourth line +	21 (11.2)
Baseline comorbidities, n (%)	
Chronic myocardial infarction	7 (3.7)
Congestive heart failure	4 (2.1)
Chronic obstructive pulmonary disease	16 (8.6)
Rheumatoid disease	5 (2.7)
Peptic ulcer disease	2 (1.1)
Mild liver disease	0 (0)
Moderate or severe liver disease	0 (0)
Peripheral vascular disease	2 (1.1)
Cerebrovascular disease	4 (2.1)
Dementia	4 (2.1)
Diabetes mellitus without complications	21 (11.2)
Diabetes mellitus with complications	8 (4.3)
Hemiplegia	0 (0)
Renal disease	19 (10.2)
Metastatic solid tumour	7 (3.7)
AIDS	0 (0)

IQR, interquartile range; AIDS, acquired immunodeficiency syndrome.

**Table 2:** Adverse events of interest.

Adverse Event	First Onset (N = 187)			Second Onset (N = 187)	Third Onset (N = 187)
	Grade 1/2, n (%)	Grade 3/4, n (%)	All, n (%)	All, n (%)	All, n (%)
Anemia	14 (7.4)	4 (2.1)	18 (9.6)	3 (1.6)	2 (1.1)
Arthralgia	18 (9.6)	1 (0.5)	19 (10.2)	4 (2.1)	1 (0.5)
Atrial fibrillation	12 (6.4)	4 (2.1)	16 (8.6)	1 (0.5)	0 (0)
Diarrhea	47 (25.1)	8 (4.3)	55 (29.4)	9 (4.8)	3 (1.6)
Hypertension	8 (4.3)	5 (2.7)	13 (7.0)	0 (0)	0 (0)
Infection	56 (29.9)	24 (12.8)	80 (42.8)	38 (20.3)	10 (5.3)
Major bleeding	1 (0.5)	6 (3.2)	7 (3.7)	0 (0)	0 (0)
Neutropenia	5 (2.7)	19 (10.2)	24 (12.8)	3 (1.6)	0 (0)
Rash	29 (15.5)	4 (2.1)	33 (17.6)	13 (7.0)	1 (0.5)
Thrombocytopenia	15 (8.0)	4 (2.1)	19 (10.2)	3 (1.6)	1 (0.5)

The percentages for grade 1–2 and grade 3–4 adverse events may not sum precisely to the overall percentage for a specific adverse event, due to rounding.

## Data Availability

The original contributions presented in this study are included in the article Further inquiries can be directed to the corresponding authors.
